# Book Reviews

**Published:** 1874-02-15

**Authors:** 


### Book Reviews.

A SYSTEM of Midwifery, including the Diseases of Pregnancy and the Puerperal State. By William Leishman, M.D., Regius Professor of Midwifery in the University of Glasgow, etc. 8vo; pp. 715. Philadelphia: Henry C. Lea, 1873.

It is difficult for the practical obstetrician to avoid looking with somewhat of distrust upon the pages of a new claimant for the honors of authority in his branch of science; and yet, in proportion as advance is made in general pathology, as well as in the practice of midwifery, must he expect to find that advance reflected in the works of those who are in position to be its exponents. In this light, we cannot but regard Professor Leishman's volume as a splendid contribution to the literature of his department of medicine, reflecting, as it does, the opinions of the advanced school of English obstetricians, and one which is destined to be, for a long time to come, an accepted work of reference in both England and America. It is written with the hand of a master and a scholar; and his conscientious statement of his views, when they are at variance with those of German and British authors, is calculated to gain for them the respect and attention which they deserve. The treatise includes a discussion of "the Diseases of Pregnancy and the Puerperal State," illustrated with plates, which are not only excellent, but novel to most of the students of this country. Some of these are, it is true, borrowed, without due acknowledgment, from the superb atlases of Schultz and Coste; but this error may be said to be condoned by the publication of others which are original.

In 1864, Professor Leishman published an essay on the mechanism of parturition, in refutation of the opinions advanced by the brilliant Naegele, on the biparietal obliquity of the fœtal cranium in normal positions. This was almost his first introduction to the profession in this country; and the complete corroboration, subsequently, of many of his views, by eminent medical authorities, has led to an expectation of further researches by the same author, which the volume before us fully justifies and satisfies.

On the subject of anæsthesia, the concluding topic of Professor Leishman's treatise, he writes in a manner which indicates that the wise administration of chloroform in labor, is rapidly meeting with favor on both sides of the Atlantic. He says: "The question of anæsthetics seems to us to stand thus: in eclampsia, and some cases of mania, and in all cases of operative midwifery, it is, without exaggeration, invaluable. In ordinary cases, it is always to be used with caution; but, if employed in small quantities, on a handkerchief, at the approach of each pain, towards the termination of the second stage, it can never do harm. It thus allays pain and assuages nervous irritability; and, in the hands of the skillful practitioner, it is a power for good, and never for evil."

We do not propose to examine, in a critical spirit, any one of several portions of the work, which indicate that the author has bestowed upon them less labor than the subjects demanded; but desire to refer merely to his consideration of the so-called "obliteration of the cervix," at the close of pregnancy.

Dr. Isaac Taylor, of New York, is said to have first put forth the statement that the cervical canal, instead of becoming obliterated, was rather increased in its long diameter, at the close of pregnancy; but we do not know of any one who has so fully demonstrated the fallacy of the received doctrines on this point, and so clearly described the exact anatomical disposition of these parts, as Dr. John Bartlett, of this city. In a paper read by him before the Chicago Society of Physicians and Surgeons, July 14th, 1873, entitled, "The Cervix Uteri, before, during, and after, Labor," the following statements are made:

"All that part of the uterine walls projecting, in vertex cases, like an inverted dome, into the vagina, and which may be felt enclosing the head, is the largely developed vaginal portions of the neck. * * * The vaginal portion of the cervix, at the time of the passage of the fœtus, is a collar of appreciable depth, not in the same plane with the vagina, but projecting into it. * * * After delivery, it (the vaginal portion of the cervix referred to above) may be felt as a flabby, floating collar, hanging from the uterus into the vagina, like a section of large intestine."

These statements have been confirmed by several observations of facts from most unexpected sources, which we have not space to detail.

All the plates, as well as the text, of Professor Leishman's work, present vividly to the eye the ancient doctrine of "obliteration." Plate No. 96 is, in fact, described as the "parturient canal completed by the *obliteration* of the os and cervix," and must have been designed solely from the imagined condition of the sexual organs, in the mind of the author. And yet, as in many recent obstetrical works, we can here also detect fugitive suggestions of error committed in this particular. He says, for example (p. 454): "In many cases, then, we are justified in passing the blades (of the forceps) within the uterus; and we apprehend that Dr. Ramsbotham's assertion is strictly correct, when he affirms that the forceps may be used in some cases in which as much as a third part of the circular margin of the os uteri can be felt; and there can be no doubt that, in a considerable number of cases, recession or retraction of the os, and especially of its anterior lip, does not occur immediately upon full dilatation, nor, it may be, for a considerable period thereafter."

We find no allusions to any of the American forceps, some of which possess, in our judgment, qualifications which render them far superior to the English instruments, both in point of facility of application and excellence of cutlery. Nor, indeed, do we find any allusion to the researches in the field of midwifery, set forth in the really meritorious works on the subject, recently published in this country.

We close by commending the book to every student and practitioner who desires to possess himself of the latest and most trustworthy information relative to the practice of eminent English obstetricians. J. N. H.

---

The Sphygmograph; its Physiological and Pathological Indications. The Essay to which was awarded the Stevens' Triennial Prize, by the College of Physicians and Surgeons, New York, April, 1873. Edgar Holden, A.M., M.D. Philadelphia: Lindsay & Blakiston, 1874.

This handsome volume, succeeding a previous essay by the author on that subject, is a complete treatise on sphygmographic technics; as an introduction to which we are made acquainted with Dr. Holden's improvements of the instrument. Varying considerably from Marey's device, —less in principle, indeed, than in construction—this apparatus is designed to supply a want in the practice of scientific physicians; and, if we can credit the statements as to its easy application and management, its reliable and durable mechanism, and its comparatively low cost—points which the description renders highly probable—the profession ought to be grateful to the author for his valuable contribution to the mechanical means of diagnosis.

As to the contents of the work, they are not far from the ideal which the

Doctor makes reference to—a complete dictionary of pulse-tracings. The great variety of sphygmographic writings, their accurate representation and description, as well as the clear style of the introductory chapters, entitle the book to a careful perusal and diligent study by every one interested in the progress of rational medicine. A more minute definition, however, of some individual pulse-traces, would have been desirable. The investigations into the action of drugs, by means of the instrument, are to be considered more as attempts in such a direction than as complete researches. Their chief value consists in showing what the sphygmograph may yet accomplish. Finally, without desire to depreciate the work, we cannot but notice the extreme tendency of the author to overestimate his results. Admitting that the tracings may be aptly compared to hieroglyphics, as regards the difficulty of interpretation, he shows great ambition to exceed the limits of usefulness of such a device. Apart, however, from any such over-estimation by the author, the treatise is a work deserving our just recommendation. H. G.

---

Manual of Midwifery; including the Pathology of Pregnancy and the Puerperal State. By Dr. Karl Schroeder, Professor of Midwifery and Director of the Lying-in Institution in the University of Erlangen. Translated into English, from the third German edition, by Charles H. Carter, B.A., M.D., B.S. Land., Member of the Royal College of Physicians, London, and Physician-Accoucheur to St. George's, Hanover Square, Dispensary. New York: D. Appleton & Co., 1873.

The translation of "Schroeder's Manual of Midwifery" brings within reach of the medical profession a valuable work. The fact that it has reached the third edition — the second one having been exhausted in less than a year — shows to what extent it is being used as a text-book in Germany. Its contents are systematically arranged, and each subject is treated in accordance with the latest scientific and physiological researches. The author has given due prominence to the use of external manipulation as a means of diagnosis and treatment of the fœtal position, a subject which is considered to be of great practical value by the German obstetricians, and which has not received the attention by the obstetricians of this country to which it is justly entitled. At the end of each chapter, reference is made to the authorities to which the author had recourse in preparing his work, and which affords a valuable index to the literature pertaining to midwifery.

The publishers have executed their part of the work quite creditably; and were we inclined to find fault, it would be with the rather small print.

